# Exploring handstand walking biomechanics and shoulder pain

**DOI:** 10.1038/s41598-026-51612-w

**Published:** 2026-07-21

**Authors:** Manuela Angioi, Nicole Hinds, Richard Twycross-Lewis, Claire Farmer, Aleksandra V. Birn-Jeffery

**Affiliations:** 1 Sport & Exercise Medicine, William Harvey Research Institute, Queen Mary University of London, London, UK; 2 School of Business and Law, St Mary’s University, Twickenham, London, UK; 3 Faculty of Arts and Creative and Industries, Middlesex University, London, UK; 4 School of Sport, Rehabilitation and Exercise Sciences, University of Essex, Wivenhoe Park, Colchester, CO4 3SQ England

**Keywords:** Balance, Gait, Aesthetic sports, Upper limb, Anatomy, Health care, Medical research

## Abstract

Handstands are a key skill in many specialist sports involving the use of upper limbs in a role usually associated with lower limbs. Handstand sports have a high incidence of pain in the shoulder, but with little understanding of handstand walking biomechanics. We aimed to explore the biomechanics of handstand walking in athletes with and without shoulder pain to assess underlying differences across whole limb function. Ten participants, who could handstand walk 6 steps, had force and positional data collected. Shoulder pain was assessed via questionnaire. Forces, centre of mass position, velocities, axial and torsional work, mechanical energy, and overall arm parameters were calculated from 237 stance phases. Two-way ANOVA assessed differences between participants with and without shoulder pain and arm (Right/Left). Participants with shoulder pain moved significantly slower (*p* = 0.005), exhibited shorter arm lengths (*p* = 0.013) and had greater net torsional work (*p* = 0.002) resulting in a lower axial–torsional work ratio. Altered handstand biomechanics in participants with pain demonstrate a more flexed position of the arm, often linked with higher joint loading. Handstand walking mechanics appear to closely resemble groucho-running enabling longer double support reducing limb loading on fatigable upper limbs. Future studies should account for confounders including disciplines, training volume, and gender, providing insight into the implications for upper limb full body loading.

**Supplementary Information:**

The online version contains supplementary material available at 10.1038/s41598-026-51612-w.

## Introduction

The handstand is a fundamental skill and an integral component across many sports and disciplines^1^ including, CrossFit, breakdancing^2^, parkour^3^, Kung Fu^4^ and circus arts^5^. The handstand is used differently depending on the discipline. Within circus arts, parkour and breakdancing, it is used as a transitional movement, to enable the individual to move to the next position^6^. In gymnastics^1^ and diving^7,8^ it involves significant balance time, using differing motor control strategies and is skill level dependent. Furthermore, within CrossFit, movements include handstand push-ups and handstand-walking^9^. Across the varied use of the handstand, there is evidence on balance control^1,10,11^, fatigue implications^12^ and handstand walking initiation^13,14^. Yet we are still unclear on walking handstand biomechanics.

Upper limb kinematics during handstand balance are comparable to lower limb kinematics, with the shoulder analogous to the hip^10,11^. However, a handstand position is less stable due to different musculoskeletal proportions^15^, smaller hand surface area and higher centre of mass (CoM)^1^. During a handstand balance, CoM deviation occurs predominantly in the sagittal plane and balance is maintained through shoulder torques and applying increased force through the fingers and wrists^10,11^. It remains unclear whether the shoulder-arm-wrist kinetic chain can be aligned similarly to the hip-knee-ankle kinetic chain, as deviations will have significant impact on joint stress. Dependent on the alignment of the ground reaction force with the limb this will have implications for fatigue, joint stress, injury and pain^16,17^. Therefore, we need to understand whether there are similarities in ground reaction force alignment in handstand walking.

Suboptimal handstand skills may explain high frequencies of upper limb injuries and pain reported within handstand disciplines^10^. Shoulder injury is highly prevalent in CrossFit athletes^18,19^, breakers^20^, parkour^21^ and circus performers^22^. Similarly, shoulder pain prevalence is high in gymnasts (22.8% of 79 gymnasts^23^) and CrossFit athletes (59.6% reported shoulder pain of 284 athletes^24^). Athletes often continue competing and training with pain^23^. Currently, there has been little research in understanding whether the upper limb, used in a lower limb movement, follows similar mechanical patterns typified by walking and running in humans. Therefore, the aim was to explore the handstand walking biomechanics of athletes with and without shoulder pain to assess for underlying biomechanical differences. The objective was to use a reduced order model representation of walking to define the whole limb mechanics during handstand walking.

## Results

Ten participants were included (female/male = 8/2). All participants were right-handed. Mean age was 28.3 ± 8.7 (mean ± SD) years, height 1.63 ± 0.08 m, body mass 57.4 ± 8.22 kg (Table 1). There were five participants in the shoulder, and no shoulder pain groups. Two reported bilateral shoulder pain, two right shoulder pain and one left shoulder pain. 244 stance phases were recorded and analysed with 7 stance phases removed as per methods section. Final analyses included 237 stance phases (131 left and 106 right arm).

**Table 1:** Participant anthropometric details.

	Gender	Mean (SD)
Age (years)	Female	29.0 (9.62)
	Male	25.5 (3.54)
	Total	28.3 (8.69)
Height (m)	Female	1.60 (0.05)
	Male	1.75 (0.08)
	Total	1.63 (0.08)
Body mass (kg)	Female	54.1 (4.85)
	Male	70.5 (3.69)
	Total	57.4 (8.22)

**Fig. 1:**
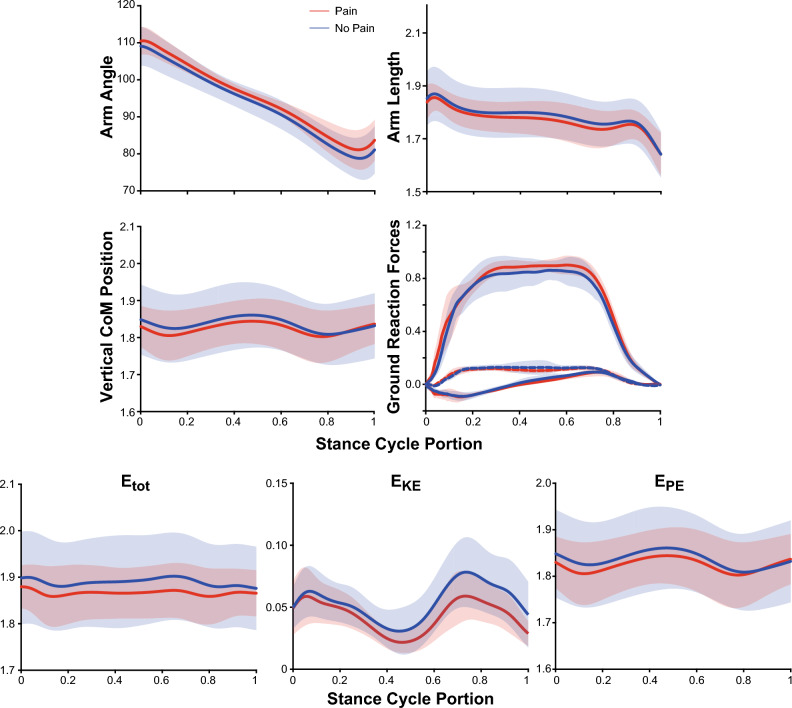
Average trajectory plots of virtual arm angle and arm length, centre of mass vertical position, the ground reaction forces and mechanical energies across athletes with and without shoulder pain over the stance phase. Shaded regions denote 95% confidence intervals of the averages. Dotted lines on the ground reaction forces denote the medio-lateral forces, to enable easier differentiation between medio-lateral and anterior–posterior forces.

Participants without shoulder pain moved significantly faster (V_a-p_: F_(1233)_ = 7.980; *p* = 0.005; ω^2^ = 0.028) and had a larger component of %W_axial_ (F_(1233)_ = 5.280; *p* = 0.022; ω^2^ = 0.013). Net torsional work (${\widehat{W}}_{tors})$ was significantly greater in those with shoulder pain (F_(1233)_ = 9.457; *p* = 0.002; ω^2^ = 0.033). Those with shoulder pain used shorter arm lengths, began stance with a more horizontally orientated arm angle and left the ground with a more upright posture (Fig. 1; Table 2). No other outcome measures significantly differed between those with and without shoulder pain (Table S1).

**Table 2:** Two-Way ANOVA results comparing handstand gait biomechanics between athletes with and without shoulder pain.

Outcome Measure	Factor	F	df	p	ω^2^
V_a–p_	**Pain**	**7.980**	**1233**	**0.005**	**0.028**
	**Arm**	**6.332**	**1233**	**0.013**	**0.021**
	Pain x Arm	0.681	1233	0.410	–
V_m–l_	Pain	0.926	1233	0.337	–
	**Arm**	**8.694**	**1233**	**0.004**	**0.032**
	Pain x Arm	1.666	1233	0.198	–
${\widehat{\mathbf{W}}}_{\mathbf{t}\mathbf{o}\mathbf{r}\mathbf{s}}$	Pain	**9.457**	**1233**	**0.002**	**0.033**
	Arm	**12.780**	**1233**	** < 0.001**	**0.045**
	Pain x Arm	**10.584**	**1233**	**0.001**	**0.037**
%W_axial_	Pain	**5.280**	**1233**	**0.022**	**0.0128**
	Arm	1.418	1233	0.235	–
	Pain x Arm	0.311	1233	0.577	–
AL_BS_	Pain	**4.449**	**1233**	**0.036**	**0.013**
	Arm	0.074	1233	0.786	–
	Pain x Arm	**22.288**	**1233**	** < 0.001**	**0.080**
AL_MS_	Pain	0.066	1233	0.798	–
	**Arm**	**5.637**	**1233**	**0.018**	**0.015**
	**Pain x Arm**	**64.752**	**1233**	** < 0.001**	**0.213**
AL_ES_	**Pain**	**5.617**	**1233**	**0.019**	**0.018**
	Arm	0.229	1233	0.632	–
	**Pain x Arm**	**20.143**	**1233**	** < 0.01**	**0.073**
AA_BS_	**Pain**	**4.876**	**1233**	**0.028**	**0.016**
	**Arm**	**4.307**	**1233**	**0.039**	**0.014**
	Pain x Arm	2.558	1233	0.111	–
AA_ES_	Pain	1.856	1233	0.174	–
	Arm	0.353	1233	0.553	–
	**Pain x Arm**	**22.033**	**1233**	** < 0.001**	**0.082**
ΔAA	Pain	0.225	1233	0.636	–
	**Arm**	**11.077**	**1233**	**0.001**	**0.039**
	**Pain x Arm**	**22.507**	**1233**	** < 0.001**	**0.083**
CoM_VertBS_	Pain	0.161	1233	0.689	–
	Arm	0.233	1233	0.630	–
	**Pain x Arm**	**59.338**	**1233**	** < 0.001**	**0.195**
CoM_VertMS_	Pain	0.519	1233	0.472	–
	**Arm**	**7.933**	**1233**	**0.005**	**0.023**
	**Pain x Arm**	**61.834**	**1233**	** < 0.001**	**0.204**
CoM_VertES_	Pain	0.391	1233	0.532	–
	Arm	0.004	1233	0.950	–
	**Pain x Arm**	**59.162**	**1233**	** < 0.001**	**0.193**

Significant findings are in bold (*p* < 0.05). ω^2^ is only reported for significant findings. Further non-significant results are presented in Table S1.

V_a-p_—anterior–posterior velocity, V_m-l_—medio-lateral velocity, , ${\widehat{{\boldsymbol{W}}}}_{{\boldsymbol{a}}{\boldsymbol{x}}{\boldsymbol{i}}{\boldsymbol{a}}{\boldsymbol{l}}}$—net axial work, ${\widehat{{\boldsymbol{W}}}}_{{\boldsymbol{t}}{\boldsymbol{o}}{\boldsymbol{r}}{\boldsymbol{s}}}$—net torsional work, %W_axial_—percentage axial work, ΔPE_step_—change in potential energy over a step, AL_BS_—arm length at begin stance, AL_MS_—arm length at mid stance, AL_ES_—arm length at end stance, AA_BS_—arm angle at begin stance, AA_MS_—arm angle at mid stance, AA_ES_—arm angle at end stance, CoM_VertBS_—vertical centre of mass position at begin stance, CoM_VertMS_—vertical centre of mass position at mid stance, CoM_VertES_—vertical centre of mass position at end stance.

## Discussion

We investigated if handstand walking biomechanics differed between participants with and without shoulder pain using an exploratory cross-sectional study design. Overall, those with shoulder pain moved slower, had greater torsional loading and altered limb posture during the stance phase (Table 2). Furthermore, handstand walking appears to reflect groucho-running gait mechanics rather than traditional walking gait mechanics (Fig. 1). Our findings are novel but due to limited sample size our results should be interpreted with appropriate caution and cannot infer causation.

Participants with shoulder pain moved slower (Table 2), which could be hypothesised as a protective response to pain. Similar findings were reported in symptomatic hip osteoarthritis patients, thus reducing joint contact forces and pain^16,25^. These patients also demonstrated reduced hip range of motion (ROM), suggesting that shoulder ROM could be reduced in participants with shoulder pain. Reduced ROM would limit shoulder flexion, reducing vertical bony alignment, and increasing torsional work, which may explain our reduced %W_axial_ and increased torsional work in those with shoulder pain (Table 2). Different hip morphologies are known to change biomechanics, with some increasing injury risk^26^. Therefore, future research should assess shoulder morphology, ROM, and biomechanics in handstand walking athletes to understand the associations with potential injury risks.

Participants without shoulder pain used greater arm lengths, and shallower arm angles (Fig. 1) during the stance phase indicating a more upright posture. An upright lower limb posture is commonly indicative of a stiffer limb, often seen in populations with pain (e.g.^27,28^) as it reduces load through the limb. However, here participants with pain used shorter arm lengths during handstand walking (Fig. 1). Humans, have significantly less, irrespective of gender and age, muscle mass on their upper limbs compared to lower limbs^29^. Handstand walking requires force generation from upper limbs, so potentially these limbs could be underpowered to generate sufficient force to compensate for pain or biomechanical deficits resulting in lower arm lengths. Additionally, patients with shoulder pathologies have poorer shoulder proprioception with significant insufficiencies in active and passive joint reposition sense^30^. It could be hypothesized that poor shoulder proprioception may instigate unintentional changes in shoulder ROM, thus additionally explaining lower limb posture in our shoulder pain participants. However, the cross-sectional study design employed herein cannot detect causality and therefore whether the pain is causing biomechanical changes or vice versa remains to be established.

Handstand walking appears mechanically similar to grounded running, also called groucho-running^31^. Spatio-temporally, the inclusion criteria required participants to utilise a double support phase—confirmed during data processing– but vertical ground reaction forces (Fig. 1) are single peaked as seen in running mechanics^32^. Maintaining a double support phase reduces peak forces, lowering limb loads which may reduce arm fatigue^10^ that could be linked with the reduced muscle mass in upper limbs^29^. Interestingly, groucho-running is a more compliant gait^31^, but our participants used a relatively small sweep angle (~ 25°; Fig. 1) potentially indicative of a stiffer gait. This stiff groucho-running gait in handstand walking may be a result of joint ROM restrictions, reduced upper limb muscle mass leading to constrained step length. However the groucho-running gait in handstand walking requires further investigation in arm ROM constraints and neuromechanical control.

Experience in a motor skill, such as handstand walking, affects the biomechanics and motor control of the movement^33,34^. Although we could not investigate this aspect within our study, our preliminary descriptive sub-analysis (Supplementary Fig. 1), suggests further research should consider both experience and training discipline.

Some limitations must be acknowledged from this study. First, the inclusion of both unilateral and bilateral pain may have introduced bias within the sample, which will limit the interpretability of the pain/no pain outcome differences. These should be taken with caution. Second, the small sample size (n = 10) limits the generalisability of the findings beyond this specific cohort and restricts population-level inference. We acknowledge that the use of all stance phases may result in pseudoreplication risking effect size overestimation due to within-subject dependence. To mitigate some of these effects we used omega squared for effect size estimation which is less sensitive to small sample sizes^35^. Due to these considerations findings should be interpreted as exploratory, and future studies with larger samples and appropriate modelling approaches (e.g., accounting for within-subject structure) are warranted to confirm these results.

Future prospective designs will help identify predisposing and consequential factors for biomechanical differences. Our sample size meant participants could not be stratified according to pain severity, experience, disciplines, age and gender. We recommend employing stratification to adjust for these confounding factors. Here, a as mentioned above a limitation of our study was the presence of unilateral and bilateral pain participants. In future work, these should be sub-grouped. In addition, more detailed characterization such as pain duration and functional status would benefit further the pathophysiological interpretation of our results. Our study is an initial exploration in handstand walking biomechanics. To understand all the complexities involved within this specialised skill, we need to embrace strategies that allow multiple disciplines, ranges of experience, age, pain and gender within a single large cross-sectional study.

## Conclusions

Handstand walking is a complex and specialised skill, used in multiple disciplines with a high prevalence of shoulder pain. In our exploratory cross -sectional study participants with shoulder pain moved significantly slower, used a shorter arm length, lower axial loading percentage and higher torsional work. This study cannot suggest causality, but these mechanical changes could be a protective response to pain. Future large cross-sectional research should account for different disciplines, training volume, pain duration and function, age and gender. Involving these confounders will provide novel understanding of handstand walking, implications for upper limb full body loading and ultimately develop strategies to enable “healthy” handstand walkers.

## Materials and methods

### Participants

Ethical approval was obtained by Queen Mary University of London Research Ethics Committee under approval reference QMREC2014/122. All methods were performed in accordance with the relevant guidelines and regulations. Inclusion criteria involved athletes ≥ 18 years, from any gender, sport, and level, who could walk minimum of 6 handstand steps and maintain shoulders, hips, and feet in vertical alignment. Exclusion criteria included any medical conditions where exercise was contraindicated. Informed written consent was collected prior to data collection.

### Procedures

Prior to biomechanics data collection, participants completed a demographics and pain questionnaire (Supplementary Material S1). Any shoulder pain during a handstand was measured using the Visual Analogue Scale (VAS)^36^. Participant height and body mass were measured using a calibrated stadiometer (Seca 271, UK) and digital weighing scales (MPMS 250, UK). Participants undertook self-selected warm up prior to data collection.

**Fig. 2:**
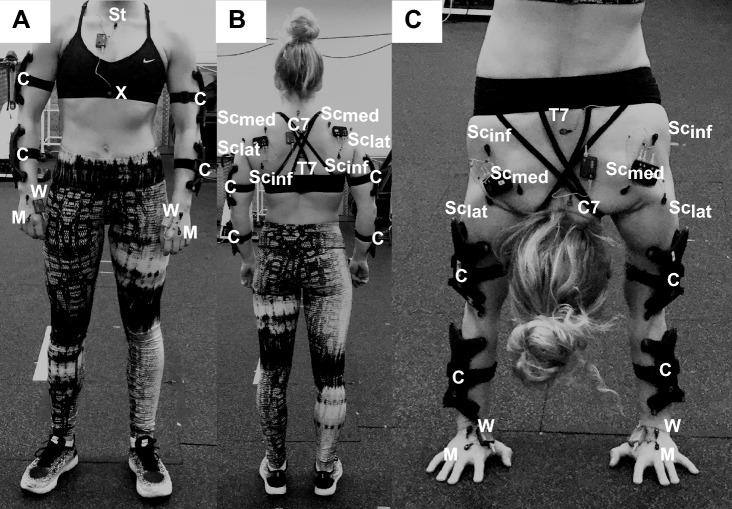
Codamotion marker setup for handstand walking; (**A**) Anterior marker setup, (**B**) Posterior marker setup, (**C**) Posterior marker setup in handstand position. Cluster sets were used to enable joint rotations to be calculated; these were located on the humerus and forearm. Individual markers denoted specific landmarks. Centre of mass was approximated using the xiphoid and T7 markers. A hand position was estimated using markers M and W. The following abbreviations in the images correspond to these landmarks; C—cluster marker, C7—7th cervical spinous process, M—proximal to 3rd metacarpophalangeal joint to enable joint flexion to occur; Scinf—inferior angle of the scapula, Sclat—lateral angle of the scapula, Scmed—medial angle of the scapula, St—sternal notch, T7—7th thoracic spinous process, W—distal to wrist joint centre to enable flexion and extension to occur unimpeded, X—xiphoid process.

Individual and cluster active infrared markers (Charnwood Dynamics Ltd, UK) were placed on the upper body (Fig. 2). Using a Codamotion Pointer virtual infrared markers were placed at the ulnar and radial styloid processes, lateral and medial humeral epicondyles and acromioclavicular joints to estimate wrist, elbow and shoulder centre of rotations.

Participants were instructed to handstand walk over two Kistler force plates (Kistler type 9281B, Switzerland) recording at 1000 Hz. Each participant started two steps in front of, and continued handstand walking two steps after, the force plates to ensure steady locomotion over the recording region. Motion capture data were recorded at 200 Hz by four calibrated 3D scanners (Codamotion CX1, Charnwood Dynamics, UK). Each participant completed five trials with the right hand on the force plates and five with the left. Trials were discounted and repeated if there was incomplete force plate hand placement or significant travel in the coronal plane (assessed visually).

### Data analysis

Data was processed using a custom written script in Matlab (version 2017a, MathWorks, USA). Stance phases were identified using ground reaction forces. Centre of mass (CoM) was estimated by averaging the T7 and xiphoid markers in the anterior–posterior and medio-lateral planes and the vertical position of the xiphoid marker. Hand position was defined by averaging the two hand markers (Fig. 2C: M and W).

For each stance phase peak vertical force (Peak *F*_vert_), anterior–posterior *(V*_a-p_), mediolateral (*V*_ml_) and vertical (*V*_vert_) velocity, axial ${\widehat{(W}}_{axial})$, torsional (${\widehat{W}}_{tors})$ and percent axial work (%W_axial_) of the virtual arm (line connecting hand position to CoM) were calculated using methods previously described^37^. Axial work is defined as the shortening and lengthening along the longitudinal axis and net torsional work is angular displacement of the virtual leg due to a perpendicular force^37^.

Energy recovery (ER_step_) and changes in potential energy (ΔPE_step_), kinetic energy (ΔKE_step_) and mechanical work (ΔMW_step_) over a step were calculated^38^. Arm length (AL) and angle (AA) in the sagittal plane and vertical centre of mass position (CoM_Vert_) were extracted at begin (_BS_), mid (_MS_) and end stance (_ES_). To allow comparison between participants, despite differences in size that are known to impact locomotion dynamics^39,40^, all outputs were rendered dimensionless using gravitational acceleration, mass (kg) and arm length (m) (calculated from height and arm span^41^).

### Statistical analysis

Descriptive characteristics were reported as means ± standard deviation (SD). All outputs were assessed for normality using Kolmogorov–Smirnov test and histogram inspection. Of the 20 biomechanical outputs, 15 were normally distributed. V_a-p_, ΔPE_step_, ΔKE_step_ and ΔMW_step_ were normalised using squareroot, Boxcox, Log10*Log10 and Log10 functions respectively. Outlier data, based on test or lab errors (i.e. poor marker visibility or incomplete stance phase), were identified and removed where present. Using SPSS Statistics (v28.0) regression analysis was performed on all outcomes against *V*_a-p_. If significant then residuals were used to negate the effect of speed^42,43^, as biomechanics of movement are known to change with speed^44^, therefore to remove this confounder speed needs to be removed from the data. Linear regression revealed V_m-l_, V_vert_, ${\widehat{\mathrm{W}}}_{\mathrm{a}\mathrm{x}\mathrm{i}\mathrm{a}\mathrm{l}}$, ΔKE_step_, ΔMW_step_, AL_BS_, AL_MS_, AA_BS_, AA_MS_, AA_ES_, CoM_VertBS_ and CoM_VertMS_ were affected by anterior–posterior velocity, therefore residuals were used in all analyses.

Two-way ANOVA tests were conducted using JASP (v0.18.3, JASP Team 2024) to assess differences between participants with and without shoulder pain and arm (R/L). Arm was included as a confounder due to both unilateral and bilateral shoulder pain participants. Significance level was set at 0.05. ω^2^ was used to report effect size as this performs better with small group sizes than eta-squared^35,45^.

## Supplementary Information

Supplementary Information 1.

Supplementary Information 2.

## Data Availability

Raw data available on request to authors.
