# Supplementary figures and images for: Exploring handstand walking biomechanics and shoulder pain
Source: Sci Rep. 2026 Jul 21;16:22766. doi: 10.1038/s41598-026-51612-w (PMC13385871; doi:10.1038/s41598-026-51612-w)

### Training Volume

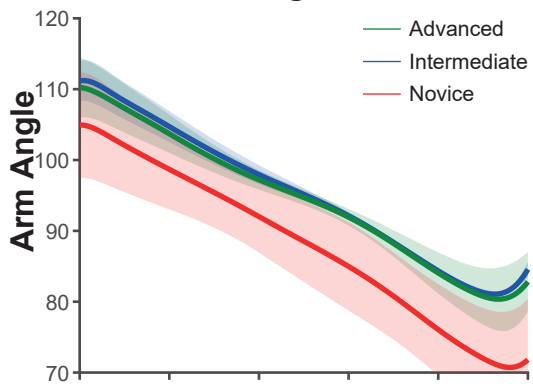

### Current Sport

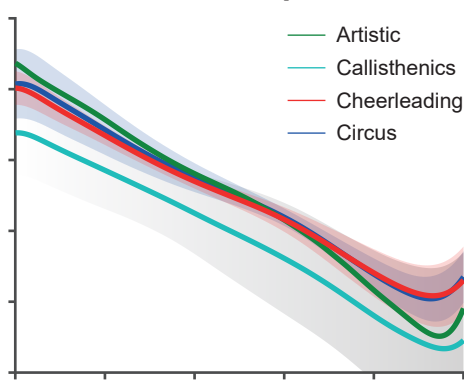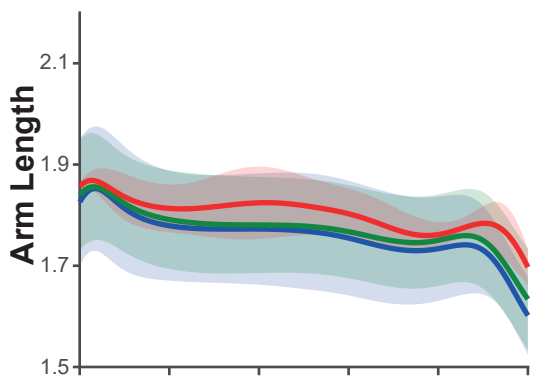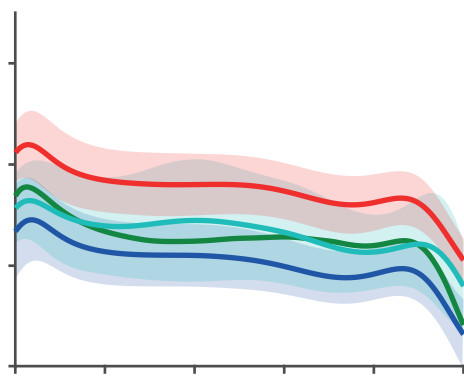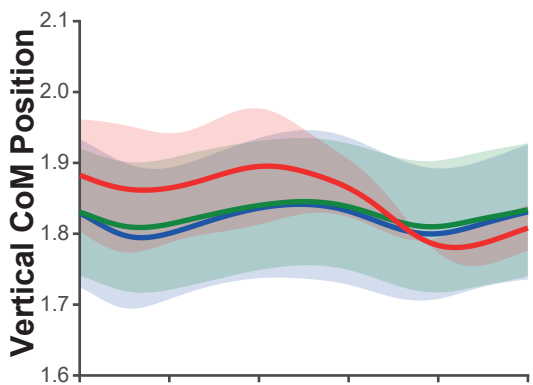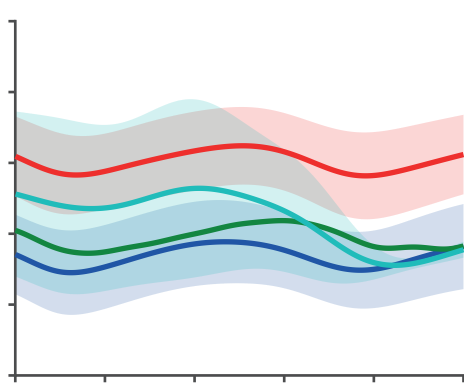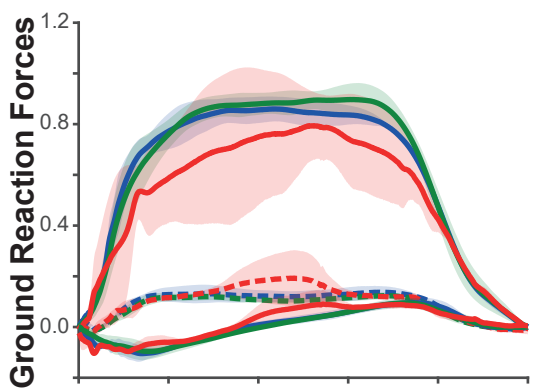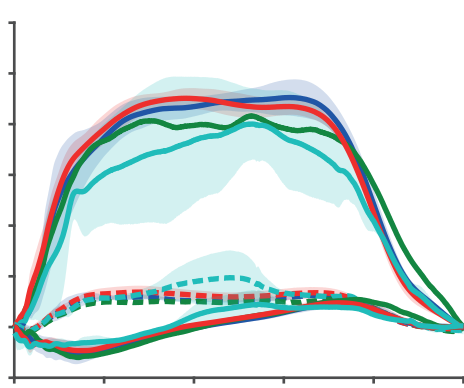

### Stance Cycle Portion

Supplement: Supplementary file 1 — Supplementary Information 1. [file 41598_2026_51612_MOESM1_ESM.pdf]
