# Supplementary material for: Exploring handstand walking biomechanics and shoulder pain
Source: Sci Rep. 2026 Jul 21;16:22766. doi: 10.1038/s41598-026-51612-w (PMC13385871; doi:10.1038/s41598-026-51612-w)
Supplement: Supplementary file 2 — Supplementary Information 2. [file 41598_2026_51612_MOESM2_ESM.docx]

**Supplementary Material**

**S1: Demographic and Pain Questionnaire**

1. Date of Birth [DD/MM/YYY]:
2. Gender [Please circle]: M F
3. What hand would you say is your dominant hand? [Please circle]: Right Left Both
4. What handstand sports have you previously participated and had formal coaching in?

[i]. Please specify in the second column below how many years in total you have participated in each sport and average number of hours per week.

Participation = at least once weekly practice/training sessions taking place on a regular basis.

Please specify exact dates e.g. 2008 to 2012 and average number of hours per week if possible.

[ii]. Please specify in the third column below how many years of formal coaching you have received in each of the sports listed.

Formal training = at least once weekly, regular training session[s] involving a qualified coach within that sport. Please also specify exact years e.g. 2008 to 2012 if possible.

| **Sport** | **4[i] Participation**  **[Years and hours per week]** | **4[ii] Formal coaching**  **[Years]** |
| --- | --- | --- |
| Gymnastics [please specify type e.g. artistic, acrobatic, rhythmic etc,] |  |  |
| Cheerleading |  |  |
| Circus Arts |  |  |
| Diving |  |  |
| Parkour/Free Running |  |  |
| Dance [please specify genre e.g. contemporary, breakdancing etc.] |  |  |
| Cross-Fit/Callisthenics training |  |  |
| Other [please specify] |  |  |

1. Do you currently experience any shoulder pain during a handstand? [Please circle]:

Y N

1. In which shoulder[s] do you experience the pain? [Please circle]:

Right Left Both

1. Please rate the severity of the shoulder pain you experience by a single vertical mark on the appropriate line below [0 = no pain, 10=worst pain imaginable].

If you only have pain in one of your shoulders, please only fill in the relevant line.

Right Shoulder Pain:

0

10

Left Shoulder Pain:

10

0

1. Do you currently have, or have you previously had any shoulder problems diagnosed by a healthcare professional [doctor, physio, osteopath etc.]? If so please specify the following for each shoulder injury:

- Diagnosis given by healthcare professional
- Date you were diagnosed
- Cause of injury
- Treatment received e.g. home exercises, physiotherapy, medications, surgery etc.

1. Do you currently have any other on-going injuries or pains? [Please specify injury location and diagnosis].
2. What is your occupation, and do you participate in any other activities on a regular basis that have a high demand on your upper body or that may affect your shoulders?

*Examples may include any manual occupation, weight training, swimming, throwing sports etc.*

**Supplementary Table S1**

| **Outcome Measure** | **Factor** | **F** | **df** | **p** | **ω^2^** |
| --- | --- | --- | --- | --- | --- |
| **PeakF_vert_** | Pain | 0.409 | 1,233 | 0.523 | - |
|  | Arm | 1.331 | 1,233 | 0.250 | - |
|  | Pain x Arm | 0.513 | 1,233 | 0.474 | - |
| **V_vert_** | Pain | 0.700 | 1,233 | 0.404 | - |
|  | Arm | 0.100 | 1,233 | 0.752 | - |
|  | Pain x Arm | 0.005 | 1,233 | 0.946 | - |
| ${\hat{\boldsymbol{W}}}_{\boldsymbol{axial}}$ | Pain | 0.410 | 1,233 | 0.523 | - |
|  | Arm | 0.608 | 1,233 | 0.436 | - |
|  | Pain x Arm | 0.076 | 1,233 | 0.783 | - |
| **ΔKE_step_** | Pain | 0.256 | 1,233 | 0.614 | - |
|  | Arm | 1.094 | 1,233 | 0.297 | - |
|  | Pain x Arm | 0.034 | 1,233 | 0.854 | - |
| **ΔMW_step_** | Pain | 0.208 | 1,233 | 0.649 | - |
|  | Arm | 1.052 | 1,233 | 0.307 | - |
|  | Pain x Arm | 0.062 | 1,233 | 0.803 | - |
| **ER_step_** | Pain | 0.344 | 1,233 | 0.558 | - |
|  | Arm | 0.282 | 1,233 | 0.596 | - |
|  | Pain x Arm | 1.273 | 1,233 | 0.261 | - |
| **AA_MS_** | Pain | 0.009 | 1,233 | 0.925 | - |
|  | Arm | 1.067 | 1,233 | 0.303 | - |
|  | Pain x Arm | 1.688 | 1,233 | 0.195 | - |

Peak*F*_vert_ – vertical peak force, *V*_vert_ – vertical velocity, $\hat{W}_{axial}$– net axial work, ΔKE_step_– change in kinetic energy over a step, ΔMW_step_– change in mechanical work over a step, ER_step_– energy recovery over a step, AA_MS –_arm angle at mid stance.

**Supplementary Figure S1 Caption**

Average trajectory plots of virtual arm angle and arm length, centre of mass vertical position and the ground reaction forces across multiple disciplines and training volume over the stance phase. Shaded regions denote 95% confidence intervals of the averages. Dotted lines on the ground reaction forces denote the medio-lateral forces, to enable easier differentiation between medio-lateral and anterior-posterior forces.
